# Translocations are induced in hematopoietic stem cells after irradiation of fetal mice

**DOI:** 10.1093/jrr/rrac078

**Published:** 2022-11-24

**Authors:** Kanya Hamasaki, Tomoko Matsumoto, John Cologne, Mayumi Mukai, Yoshiaki Kodama, Asao Noda, Nori Nakamura

**Affiliations:** Department of Molecular Biosciences, Radiation Effects Research Foundation, 5-2 Hijiyama-Park, Minami-ku, Hiroshima 732-0815, Japan; Department of Molecular Biosciences, Radiation Effects Research Foundation, 5-2 Hijiyama-Park, Minami-ku, Hiroshima 732-0815, Japan; Department of Statistics, Radiation Effects Research Foundation, 5-2 Hijiyama-Park, Minami-ku, Hiroshima 732-0815, Japan; Department of Molecular Biosciences, Radiation Effects Research Foundation, 5-2 Hijiyama-Park, Minami-ku, Hiroshima 732-0815, Japan; Department of Molecular Biosciences, Radiation Effects Research Foundation, 5-2 Hijiyama-Park, Minami-ku, Hiroshima 732-0815, Japan; Department of Molecular Biosciences, Radiation Effects Research Foundation, 5-2 Hijiyama-Park, Minami-ku, Hiroshima 732-0815, Japan; Department of Molecular Biosciences, Radiation Effects Research Foundation, 5-2 Hijiyama-Park, Minami-ku, Hiroshima 732-0815, Japan

**Keywords:** radiation, fetal hematopoietic stem cell (f-HSC), chromosome aberration, multicolor fluorescence *in situ* hybridization (mFISH), mouse

## Abstract

Although mammalian fetuses have been suggested to be sensitive to radiation, an increased frequency of translocations was not observed in blood lymphocytes from atomic bomb (A-bomb) survivors who were exposed to the bomb in utero and examined as adults. Since experiments using hematopoietic cells of mice and rats confirmed this finding, it was hypothesized that either irradiated fetal hematopoietic stem cells (f-HSCs) cannot generate exchange-type chromosomal aberrations or cells bearing induced aberrations are eliminated before the animals reach adulthood. In the present study, pregnant mice (12.5–15.5 days post coitum [dpc]) were irradiated with 2 Gy of X-rays and long-term HSCs (LT-HSCs) were isolated 24 h later. Multicolor fluorescence *in situ* hybridization (mFISH) analysis of LT-HSC clones proliferated *in vitro* showed that nine out of 43 (21%) clones from fetuses and 21 out of 41 (51%) clones from mothers bore translocations. These results indicate that cells with translocations can arise in mouse f-HSCs but exist at a lower frequency than in the mothers 24 h after X-ray exposure. Thus, it seems likely that translocation-bearing f-HSCs are generated but subsequently disappear, so that the frequency of lymphocyte translocations may decrease and reach the control level by the time the animals reach adulthood.

## INTRODUCTION

The embryonic period can be divided into three primary stages according to the phase of development—preimplantation, organogenesis and fetal—and sensitivity to ionizing radiation differs among these phases [1]. When considering such complex radiosensitivity in fetuses, including postnatal cancer risk after in utero exposure, the Oxford Survey of Childhood Cancers (OSCC), a well-known epidemiological report, is often cited. OSCC, conducted in the 1950s in the UK, reported that children born to mothers exposed to diagnostic abdominal X-rays (with doses of approximately 10 mGy) during pregnancy had an increased risk of childhood leukemia and solid cancer with an approximate relative risk (RR) of 1.3–1.5 [2,3], which implies that the RR could be as high as 50 per Gy if a linear dose response was assumed. The OSCC results were subsequently confirmed by other studies, and Wakeford, in the latest review of childhood cancer following fetal radiation exposure, urged that we should not ignore the risk of childhood cancer due to low-dose medical exposure in pregnant women [4]. However, some reports do not support these OSCC results. For example, animal studies have shown that the risk of cancer after exposure in utero is often lower than that during the juvenile period [5,6]. Additionally, unlike the OSCC results, previous studies of an in utero cohort of atomic bomb (A-bomb) survivors have not shown an extremely high risk of cancer after prenatal exposure, not only for childhood cancers but also for cancers that develop in adulthood [7–12]. Therefore, the risk of cancer associated with prenatal exposure in humans remains unclear.

To provide biological and mechanistic explanations for responses following fetal exposure to radiation, we have previously examined that A-bomb survivors exposed in utero for translocation frequencies in peripheral blood lymphocytes at about 40 years of age using the G-banding method and found that the frequency did not increase with increasing radiation dose except for a possible small hump at low doses (e.g. below 0.1 Gy, and this small hump was recently reanalyzed by Cologne *et al.* [13]), while their mothers showed a clear dose-related increase in translocation frequencies [14]. This lack of dose–response in the frequency of chromosome aberrations in lymphocytes and bone marrow (BM) cells following fetal irradiation was confirmed in subsequent studies using mice [15] and rats [16]. In addition, we have reported that the effect of in utero exposure differs between tissues (hematopoietic versus non-hematopoietic cells), and that the radiation effect varies with the time of exposure, whether before or after organogenesis [17]. Based on these observations, we hypothesized that for cytogenetically aberrant cells to survive and persist, radiation damage should occur after fetal stem cells settle into their niche [18]. In the present study, we aimed to examine part of this hypothesis using hematopoietic stem cells (HSCs). During fetal development, hematopoiesis occurs first in the yoke sac at embryonic day 7.5 (E7.5), followed by secondary hematopoiesis in the aorta-gonad-mesonephros (AGM) region starting at approximately E10. Subsequent hematopoiesis occurs mainly in the liver. It is not until several weeks after birth that the HSCs finally settle into their niche in the BM [19,20]. In this respect, the microenvironments in which fetal and adult HSCs are placed are quite different. Our previous study showed that the translocation frequency in BM cells declined quickly following fetal exposure to radiation and reached the control level one week after birth [15]. In the present study, we irradiated pregnant mice with 2 Gy of X-rays at 12.5–15.5 dpc and sacrificed them 24 h later to collect LT-HSCs. Each isolated LT-HSC derived from a mother or mixed fetuses was clonally expanded *in vitro* to determine whether exchange-type aberrations were induced and detectable using the multicolor fluorescence *in situ* hybridization (mFISH) method. We compared the aberration frequency between mother and fetus to evaluate the radiation effects on LT-HSCs.

## MATERIALS AND METHODS

### Mice and irradiation

Pathogen-free B6C3F1 mice (6–8 weeks old, both male and female), the same strain used in our previous studies [15,17], were purchased from the Experimental Animal Co. (Hiroshima, Japan). Approximately two weeks after their arrival, mice were mated, and pregnant females at day 12.5–15.5 (equivalent to the fetal stage) after mating were irradiated with 2 Gy X-rays to the whole body. Irradiation was performed using a Faxitron CP-160 machine (Tucson, AZ, USA) operating at 160 kVp, 6.3 mA with a 0.5 mm aluminum and 0.21 mm copper filter at a dose rate of 0.77 Gy/min. The irradiation was performed under conditions similar those used in our previous studies, i.e. high dose and dose rates [15,17]. We examined a total of 11 pregnant mice, including 111 fetuses. All mice were kept at an animal facility in the Radiation Effects Research Foundation (RERF; Hiroshima, Japan), housed in sterile cages placed in a microisolator, and fed a sterile regular diet ad libitum. This study was approved by the Institutional Experimental Animal Care Committee (approval number: 2017–03).

### Antibodies

Antibodies were purchased from BioLegend (San Diego, CA, USA) and Invitrogen (Waltham, MA, USA). Biotin-and PE-Cy7-labeled antibodies such as anti-CD3e (145–2C11), anti-CD45R/B220 (RA3-6B2), anti-Ter-119 (TER-119), anti-Ly-6G/Ly-6C (Gr-1, RB6-8C5) and anti-CD11b (M1/70) were used. Other antibodies used were FITC-labeled anti-CD34 (RAM34), PE-labeled anti-Ly6A/E (Sca1, D7), PerCp-Cy5.5-labeled anti-CD150 (SLAM, TC15-12F12.2), APC-labeled anti-CD117 (c-kit,2B8) and APC-Cy7-labeled anti–CD48 (HM48–1).

### Cell preparation

One day after irradiation, pregnant mice were anesthetized by inhalation of isoflurane (FUJIFILM, Osaka, Japan) and euthanized by cervical dislocation, after which HSCs were collected.

### Fetuses

The liver organs of fetuses in each uterus were removed, combined, minced with a scalpel and then dispersed into single-cell suspensions in Iscove’s modified Dulbecco’s medium (IMDM; Sigma-Aldrich, St. Louis, MO, USA) supplemented with 2% FBS (Sigma-Aldrich). Due to extremely low liver stiffness in fetuses, dispersion was achieved simply by using a syringe with a 21 G needle. HSC enrichment was then performed in advance for efficient isolation by a cell sorter. After blocking the Fc-mediated reaction with CD16/32 (93) antibody (Invitrogen), the cells were stained with biotin-labeled CD3e, CD45R/B220, Ter–119 and Ly-6G/Ly-6C, which are associated with the blood cell lineage fraction. The lineage-negative cells, that is, the HSC-enriched fraction, were separated using IMagnet™ and BD IMag™ Streptavidin Particles Plus-DM (BD Biosciences, Franklin Lakes, NJ, USA). For LT-HSC identification by flow cytometry, the cells were stained with fluorescence-labeled antibodies following staining with Fixable Viability Dye eFluor 506 (Invitrogen) to detect dead cells. This combination of antibody staining has been previously described in the literature [21].

### Mothers

Hematopoietic cells were prepared from the removed femora, tibiae and spine. The bones were finely crushed with a pestle and suspended in 1× PBS. Cells were obtained by passing through a 70-μm cell strainer (Corning, Corning, NY, USA) and used for the HSC enrichment process. In the case of mothers, after Fc-mediated blocking, biotin-labeled anti-CD11b was added to the lineage fractions, as described above for the fetuses. In some experiments, CD117 positive cells were enriched using magnetic beads and auto-MACS (Miltenyi Biotec, Bergisch Gladbach, Germany). LT-HSC identification using flow cytometry after the detection of dead cells was also based on the literature [22].

### LT-HSC sorting and culture

To obtain LT-HSCs, each fetal ((Lineage^−^Sca1^+^c-kit^+^(L^−^S^+^K^+^) CD150^+^CD48^−^) and mother’s (CD34^−/low^ L^−^S^+^K^+^ CD150^+^CD48^−^) hematopoietic cell population was sorted using a cell sorter (JSAN II, Bay Bioscience, Kobe Japan, or FACS Aria II, BD Biosciences). These cell fractions are thought to contain a large proportion of LT-HSCs that are the least differentiated and retain their self-renewal ability for a long time. HSCs were isolated into wells of a 96-well round bottom plate (Corning) at one cell per well to obtain clones from single cells. To maintain humidity during cell culture, cells were aliquoted into a maximum of 60 interior wells of the plate, leaving 36 wells on the periphery to be used for the application of water only. In the present study, we conducted 11 experiments in total (i.e. 11 mothers and their 111 fetuses were used), and LT-HSCs were isolated in each experiment. As the liver of each fetus born to a mother is too small, the cells from multiple fetuses were mixed to achieve the requisite sample quantity. Moreover, the number of isolated LT-HSCs varied based on the experiment (i.e. the range was 16–180 LT-HSCs, and 895 cells were obtained from the fetuses; the range was 14–240 LT-HSCs, and 1391 cells were obtained from the mothers). The isolated LT-HSCs were cultured in MethoCult GF M3434 medium (Stem Cell Technologies, Vancouver, Canada), which was specifically designed to support hematopoietic colonies, such as Burst-Forming Unit-Erythroid (BFU-E), Colony-Forming Unit-Granulocyte, Macrophage (CFU-GM) and Colony-Forming Unit-Granulocyte, Erythroid, Macrophage, Megakaryocyte (CFU-GEMM). Cultures were run for approximately 7–10 days at 37°C in a 5% CO_2_ incubator. Hematopoietic colonies that proliferated during culture were thought to be derived from single LT-HSC with colony forming ability. An additional experiment was conducted to verify the accuracy of LT-HSC sorting. Sorted LT-HSCs were cultured in a medium that only allowed the growth of more primitive stem cells, such a medium includes; F12 (Sigma-Aldrich), 1% ITSX (Gibco, Waltham, MA, USA), 1% P/S (FUJIFILM), 10 mM HEPES (FUJIFILM), TPO (PeproTech Cranbury, NJ, USA), SCF (PeproTech) and PVA (Sigma-Aldrich) [23]. Initial culture in the medium was maintained for several days in a fibronectin-coated 96 well plate (Corning). The expanded cells were then transferred into a 24-well plate in MethoCult GF M3434 medium to allow further proliferation.

### Cell harvest and mFISH karyotyping

**Fig. 1:**
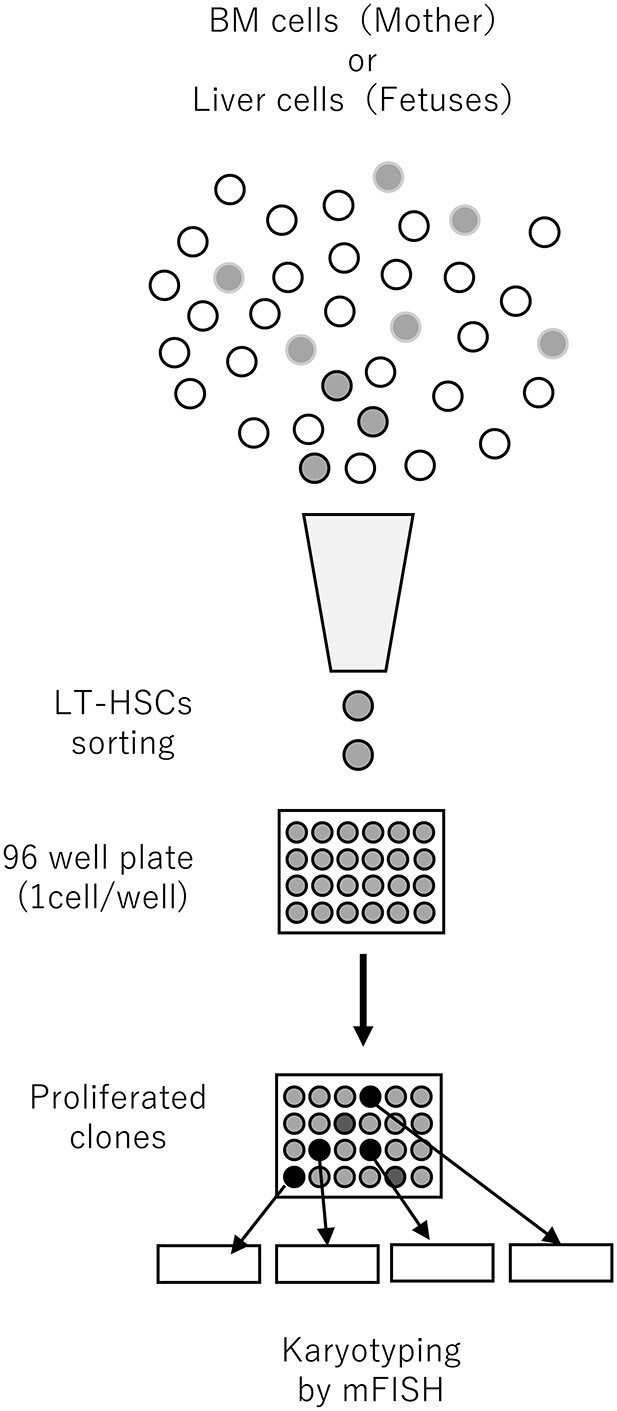
Schematic illustration of the process of LT-HSC sorting, culture and karyotyping.

Each expanded clone was harvested for chromosomal testing. Colcemid (100 ng/ml; Serva Electrophoresis, Heidelberg, Germany) or metaphase arresting solution (100× dilution; Genial Helix, Chester, UK) was added to suppress spindle formation during the last 18 h of culture. To perform hypotonic treatment the cells were suspended in hypotonic solution (0.075 M KCL:1% sodium citrate solution, 3:1) for 15 min at 37°C. Fresh fixative (3:1 methanol: acetic acid) was then added to the tube containing the hypotonic solution (half-fix). After 15 min, the fixative was replaced once or twice. Chromosome slides were prepared by a standard air-drying method [15]. Metaphase chromosomes were hybridized with a set of chromosome probes (mFISH probes) according to the manufacturer’s instructions (21XMouse probe, MetaSystems, Altlussheim, Germany). The resulting mFISH images were analyzed with a Genus/Chromofluor System (Applied Imaging International, New Castle upon Tyne, UK)) and Isis/mFISH software (MetaSystems). The mFISH software can be used to detect exchange-type chromosomal aberrations such as translocations and insertions for all chromosomes. We examined the karyotypes of only a few metaphases from each clone because the observed karyotypes were the same within each clone, given that each clone was derived from a single LT-HSC. The flow of the study procedures, from cell sorting to karyotyping, is shown in Fig. 1.

### Statistical methods

Multiple aberrations per clone were rare in mothers and non-existent in clones derived from fetuses. In particular, multiple translocations in mothers did not occur when translocations involving the X chromosome were excluded. Therefore, we treated the number of aberrations as a binomial variable, with the number of clones as the denominator. We used a binomial proportions test to compare translocation frequency between mother and fetus clones, and based on previous results showing a lack of translocation induction in fetuses, we chose to perform a one-sided test.

## RESULTS

### Translocations are induced in fetal HSCs

In this study, we isolated LT-HSCs from each irradiated mother and corresponding fetuses. Among them, only cells that had the ability to form colonies were able to proliferate. Finally, we found that nine HSC clones out of 43 (21%) derived from irradiated fetuses bore translocation that could be inferred as directly caused by exposure to radiation. This indicates that rejoining of broken chromosomes can occur in f-HSCs and that translocation-bearing stem cells can persist for at least 24 h after exposure to radiation. However, the frequency of translocations was lower than that observed in irradiated mothers (21 out of 41 clones: 51%, *P* = 0.004). Also, no aberrations were observed in non-irradiated fetuses (15 clones) and non-irradiated mothers (12 clones).

### Verification that the isolated clones are LT-HSCs

Among BM stem cells, hematopoietic progenitor cells (HPCs) are more abundant than HSCs. Consequently, although we believe that the surface markers used for sorting of LT-HSCs with fluorescence activated cell sorting (FACS) are legitimate [21,22] and that FACS processes were properly performed, it is possible that some HPCs were retained despite sorting and that the MethoCult GF M3434 medium might have supported their growth. To exclude the possibility that the isolated clones were mostly HPCs rather than HSCs, we used the defined, albumin-free culture medium recently developed for the long-term expansion of HSCs ex vivo [23] and cultured the FACS-sorted cells (expected to be LT-HSCs) for one week. Subsequently, the culture medium was replaced with MethoCult GF M3434 medium for further expansion of clonal cells for mFISH analysis. Among the nine clones obtained from fetuses, two were found to carry translocations (22%). This frequency is close to that observed in the main results mentioned above (i.e. nine out of 43; 21%). Thus, it seems likely that translocations were induced and present in the f-HSCs.

### Skewed distribution of the chromosomes involved in translocations

When we examined the translocations observed closely, skewed distributions were observed. First, among the translocations derived from irradiated fetuses, eight clones out of nine were found to have originated from male fetuses (chromosomally XY), and the remaining clone had an indeterminate sex origin, as the chromosome constitution was XO (Table 1). Second, among the clones derived from the irradiated mothers, nine exchange-type chromosomal aberrations out of 21 (43%) involved X chromosomes, a frequency approximately four times higher than the expected frequency based on the assumption of random distribution of translocation breakpoints among chromosomes. Thus, additional analysis that excluded abnormalities involving the X chromosomes did not provide a strong evidence of differences between mothers’ and fetuses’ translocation frequencies (*P* = 0.09), although without translocations involving the X chromosome the proportion of translocations in mothers’ clones (37%) was still almost twice as large as that in the clones derived from fetuses. The reasons for the preponderance of aberrant clones occurring in male fetuses or involving the X chromosomes of maternal HSCs are unclear. Regarding the latter, we performed XIST/Xcen/Xtelo FISH to determine whether the maternally inactive X chromosome was exclusively involved in translocation. Although the data are limited, the results did not indicate the exclusive involvement of inactivated X chromosomes (see Supplementary Data).

**Table 1:** Abnormal karyotypes identified by mFISH

**Fetuses**	**Mothers**
39,X,(−X or -Y),t(5q±;6q±)	40,XX,t(13q-;15q+)
40,XY,t(15q-;17q+)	40,X,t(Xq±;15q±)
40,XY,t(7q+;14q-)	40,X,t(Xq+;12q-)
40,XY,t(2q-;7q+)	40,t(Xq+;Xq-),t(2q-;17q+)
40,XY,t(10q+;16q-)	40,XX,t(8q+;10q-)
40,XY,t(11q-;16q+)	40,XX,t(9q+;15q-)
40,XY,t(5q-;18q+)	40,XX,t(8q-;13q+)
40,XY,t(1q+;5q-)	39,-X,t(Xq+;2q-),t(1q±;15q±)
40,XY,t(9q+;10q-)	40,X,t(Xq-;2q+)
	40,XX,t(2q-;7q+),del(8q)
	40,X,t(Xq-;7q+),t(16q-;18q+)
	40,X,ins(4q+;Xq-)
	40,XX,t(10q+;13q-)
	40,XX,t(1q-;12q+)
	40,X,t(Xq-;6q+)
	40,XX,t(2q-;10q+)
	40,XX,t(6q+;6q-)
	40,X,t(Xq-;13q+)
	40,XX,t(4q-;6q+)
	40,XX,t(8q+;11q-)
	40,XX,ins(6q+;16q-)

## DISCUSSION

Our previous studies indicated that the hematopoietic system is exceptional in not recording the radiation damage incurred during fetal life [14–17]. We hypothesized that this is because f-HSCs are not yet located in their proper microenvironment (niche) in the BM, which is established several weeks after birth, and that aberration-bearing cells are somehow excluded from entering the BM niche.

Two possibilities were proposed. First: (i) no repair of radiation-induced DNA double-strand breaks can take place in fetal HSCs that are not yet in their final niche in the BM, so the damaged cells would remain arrested in the cell cycle and will be finally diluted out or subjected to apoptotic death (no repair model). Supportive evidence for this model is that expression of the ATM gene, which is an essential gene in DNA damage response, is at a low level in HSCs in E14.5 fetal liver [24]. Alternatively, (ii) DNA damage repair may take place in fetal HSCs, but the translocation-bearing cells are somehow lost either soon after the exposure or at a later postnatal stage when they enter the BM niche (negative selection model) [18].

In this study, we showed that DNA repair of radiation-induced cell damage occurs in f-HSCs at least within 24 h of radiation exposure, but the frequency of translocations produced by misrepair is lower than that in the mothers. Therefore, the present results support the latter (negative-selection) hypothesis. However, these results should be carefully considered. According to the study by Wang *et al.* [25], the number of high-dose-irradiated BM cells (including HSC and HPC) rapidly decreases 1–3 days after exposure. Similar to this, we have experienced the problem that the number of HSCs obtained was much lower on the second day after exposure than on the first day. This means that one day after exposure, the removal of radiation-damaged cells is still in progress. Thus, it is possible that the cell culture *in vitro* of sorted LT-HSC partially rescues the cells that would have been eliminated *in vivo*. Although such a possibility exists, if translocations were induced in fetal LT-HSCs following misrepair of radiation-induced chromosome breaks and persist *in vivo*, then how would it be possible for the translocation-bearing cells to be deleted from the HSC pool? Simple mechanical models are not applied here because stable-type aberrations do not pose a problem for continued cell division.

One possible explanation for the present and past observations is that translocation-bearing HSCs might have slightly inferior growth capability compared to their normal counterparts. Although no sign of this was observed during clone isolation *in vitro*, a slightly longer cell cycle time might effectively exclude translocation-bearing cells *in vivo*.

Another possibility is that the BM niche might be able to distinguish normal HSCs from aberrant ones and accept only normal and healthy HSCs. For example, translocation-bearing cells must have undergone a DNA damage response, which could induce inflammatory substances, such as NF-κB [26]. Cells that compose the BM niche may be able to sense such a condition and refuse the damaged cells from entering the niche. In this regard, it is interesting to note that in mice, prior to 4 weeks of age, cycling HSCs are not allowed to enter the BM niche. A CXC chemokine ligand, CXCL12, appears to be a factor related to this observation [27].

A third possibility, not mutually exclusive from the above two, is that fetal HSCs that had undergone a DNA damage response might not be able to remain in the stem cell line, shifting instead toward more differentiated lineages. This idea is based on our observation that the translocation frequency is always lower in BM cells than in spleen cells following fetal irradiation in mice [15], and mice irradiated as fetuses sometimes develop clonal translocations in their lymphocytes while the translocation frequency is close to the control level [28]. It has also been indicated that BM stem cells can differentiate into non-lymphoid cells, such as male germ cells [29], oocytes [30], or neuronal cells [31], although the results are still arguable [32]. However, this idea needs to be recognized when considering the development of childhood leukemia. As the majority of childhood leukemias are acute lymphoblastic leukemia (ALL), and the target in ALL is B progenitor cells, even if abnormal cells generated in HSCs are relegated to B progenitor cells, they might become malignant in that state.

In conclusion, when f-HSCs are exposed to radiation, translocations occur as in the mothers, but the frequency in fetuses is lower 24 h after irradiation. Further studies are needed to determine whether the subsequent process of further disappearance of f-HSCs bearing chromosomal abnormalities is dependent on events, such as cell competition. It is also important to track the transition of translocations resulting from in utero exposure and demonstrate their relationship with childhood leukemia. Successfully addressing these issues may resolve the long-standing debate on the effects of in utero exposure vis-à-vis the risk of childhood leukemia.

### PRESENTATION AT A CONFERENCE

A part of this work was presented in a poster at 16^th^ International Congress of Radiation Research 2019.

## FUNDING

The Radiation Effects Research Foundation (RERF), Hiroshima and Nagasaki, Japan is a public interest foundation funded by the Japanese Ministry of Health, Labour and Welfare (MHLW) and the US Department of Energy (DOE). This publication was supported by RERF Research Protocol RP-P4–17. This work was supported in part by JSPS KAKENHI Grant Number. JP16K00556. The views of the authors do not necessarily reflect those of the two governments.

## Supplementary Material

Revise-Hamasaki_Supplementary_Data_rrac078
