# Supplementary material for: Translocations are induced in hematopoietic stem cells after irradiation of fetal mice
Source: J Radiat Res. 2022 Nov 24;64(1):99–104. doi: 10.1093/jrr/rrac078 (PMC9855322; doi:10.1093/jrr/rrac078)
Supplement: Revise-Hamasaki_Supplementary_Data_rrac078 [file revise-hamasaki_supplementary_data_rrac078.docx]

Supplementary data

Summary of XIST/Xcen/Xtelo FISH study data and methods

| Clone | Number of cells analyzed | Average distance between the two signals (telomere and centromere) | | Karyotype |
| --- | --- | --- | --- | --- |
|  |  | In active X chromosome (µm) | In inactive X chromosome (µm) |  |
| X-1 | 18 | 3.3 | 1.8 | Normal |
| X-2 | 17 | 3.8 | 1.8 | Normal |
| X-3 | 47 | 6.6 | 1.7 | t(Xq-;9q+) |
| X-4 | 128 | 3.1 | 1.8 | t(Xq+;8q-) |
| X-5 | 22 | 4.1 | 1.9 | Normal |
| X-6 | 12 | 2.7 | 2.0 | Normal |
| X-7 | 33 | 2.5 | 1.5 | t(Xq-;18q+),t(1q-;2q+) |
| X-8 | 24 | 3.0 | 1.4 | t(3q+;4q-) |
| X-9 | 21 | 4.3 | 2.5 | Normal |
| X-10 | 17 | 3.1 | 1.6 | Normal |
| X-11 | 12 | 3.8 | 1.9 | Normal |
| X-12 | 23 | 3.4 | 1.5 | Normal |

Method

According to the instructions, slides that were cytospun and fixed with 4% PFA were hybridized with a set of mouse XIST/Xcen/Xtelo FISH probes (Chromosome Science Labo Inc. Sapporo, Japan) overnight at 37 °C. After washing with 50% formamide/2xSSC the next day, DAPI staining (250 ng/ml) was performed. Observations were obtained with an Axio Imager Z2 microscope (Zeiss, Jena, Germany), and the distance in the signal from Xcen (Cy3-red) to Xtelo (Cy5-yellow) in both X chromosomes was measured using Isis/mFISH software.

We can easily distinguish between inactive (Xi) and active (Xa) X chromosomes using this approach, because only two signals (red and yellow) are observed in Xa, whereas an untranslated RNA region (FITC green) is also observed in Xi (in addition to red and yellow signals). The untranslated RNA region is a transcription product of *XIST* which is required for X chromosome inactivation, and the XIST signal can be observed covering an Xi.

We performed XIST/Xcen/Xtelo FISH to determine whether the inactive maternal X chromosome exclusively participates in translocations involving X chromosomes. Depending on the arrangement of each chromosome in the cell nucleus, if translocation occurs between an X chromosome and other chromosomes, the distance between the Xcen and Xtelo signals in the X chromosome of the clone might change. Therefore, we compared the distances between the Xcen and Xtelo signals among 12 clones (eight normal karyotypes, three translocations involving an X chromosome, one clone with translocation involving other chromosomes). These 12 clones were obtained by additional experiments, and karyotyping was performed using mFISH.

Results

First, there were no clones in which the Xcen or Xtelo signals were far outside the XIST region in the inactive X chromosome among the three clones involving an X chromosome translocation (X-3, X-4, and X-7). No clones were observed that clearly suspected translocation involving inactive X chromosome. Next, only clone X-3 (indicated in gray in the above table) exhibited a notable change in distance, and the change was in the active, not the inactive, X chromosome. The distance was 6.0 µm in the Xa of clone X-3, compared with average distance 3.6 µm (SD 0.53) in the Xa of 9 clones not involving an X chromosome translocation; the normal tail probability of such an observed difference from the overall mean is < 0.01. On the other hand, the distance was 1.7 µm in the Xi of clone X-3, compared with average distance 1.9 µm (SD 0.30) in the Xi of 9 clones not involving an X chromosome translocation; the normal tail probability of such an observed difference from the overall mean is 0.56. Therefore, we concluded that the active X chromosome, not the inactive X chromosome, was involved in the translocation of clone X-3, and we therefore ruled out the possibility that only inactive maternal X chromosomes participated in translocations involving an X chromosome.
